# Enhancement of Carbon Sequestration in Soil in the Temperature Grasslands of Northern China by Addition of Nitrogen and Phosphorus

**DOI:** 10.1371/journal.pone.0077241

**Published:** 2013-10-10

**Authors:** Nianpeng He, Qiang Yu, Ruomeng Wang, Yunhai Zhang, Yang Gao, Guirui Yu

**Affiliations:** 1 Key Laboratory of Ecosystem Network Observation and Modeling, Institute of Geographic Sciences and Natural Resources Research, CAS, Beijing, China; 2 State Key Laboratory of Forest and Soil Ecology, Institute of Applied Ecology, CAS, Shenyang, China; 3 Department of Biology, Graduate Degree Program in Ecology, Colorado State University, Fort Collins, Colorado, United States of America; 4 State Key Laboratory of Vegetation and Environmental Change, Institute of Botany, CAS, Beijing, China; University of Maryland, United States of America

## Abstract

Increased nitrogen (N) deposition is common worldwide. Questions of where, how, and if reactive N-input influences soil carbon (C) sequestration in terrestrial ecosystems are of great concern. To explore the potential for soil C sequestration in steppe region under N and phosphorus (P) addition, we conducted a field experiment between 2006 and 2012 in the temperate grasslands of northern China. The experiment examined 6 levels of N (0–56 g N m^-2^ yr^-1^), 6 levels of P (0–12.4 g P m^-2^ yr^-1^), and a control scenario. Our results showed that addition of both N and P enhanced soil total C storage in grasslands due to significant increases of C input from litter and roots. Compared with control plots, soil organic carbon (SOC) in the 0–100 cm soil layer varied quadratically, from 156.8 to 1352.9 g C m^-2^ with N addition gradient (R^2^ = 0.99, *P* < 0.001); and logarithmically, from 293.6 to 788.6 g C m^-2^ with P addition gradient (R^2^ = 0.56, *P* = 0.087). Soil inorganic carbon (SIC) decreased quadratically with N addition. The net C sequestration on grassland (including plant, roots, SIC, and SOC) increased linearly from -128.6 to 729.0 g C m^-2^ under N addition (R^2^ = 0.72, *P* = 0.023); and increased logarithmically, from 248.5 to 698 g C m^-2^under P addition (R^2^ = 0.82, *P* = 0.014). Our study implies that N addition has complex effects on soil carbon dynamics, and future studies of soil C sequestration on grasslands should include evaluations of both SOC and SIC under various scenarios.

## Introduction

Human activity has doubled the atmospheric deposition of nitrogen (N) over the past century [1,2]. N deposition can influence the biogeochemical coupling of the carbon (C) and N cycles in soil by altering organic matter decomposition [3-5], belowground C allocation [6,7], and microbial communities and activity [8,9]. However, it remains unclear whether N limits soil C sequestration in terrestrial ecosystems, although the promotion of plant growth and ecosystem primary production are observed in many regions [3,6,7]. The effect of N addition on soil C sequestration is a concern because soil C sequestration in terrestrial ecosystems is an important approach to offset anthropogenic CO_2_ emissions [10].

Increases in N deposition have been predicted to increase terrestrial C storage [11,12], but the results of field experiments were inconsistent. Zeng et al. (2010) reported that N addition decreased the C storage of soil and aboveground content in ecosystems to some extent [13]. On the basis of a meta-analysis of 257 studies, Lu et al. (2011) found that N addition had no apparent effect on soil C storage in either the organic horizon or mineral soil in grasslands, despite substantial increases in C inputs from roots and litter [14].

Grasslands in northern China (approximately 150 million ha) have enormous capacity to sequester atmospheric CO_2_ through good management of land use, especially via grazing-exclusion, mowing, and conversion from farmland to grassland [15-18]. Soil acidification or pH decrease was predicted in semi-arid regions with increasing N input, which might result in CO_2_ emission from dissolution of soil inorganic carbon (SIC: carbonate minerals, such as calcium carbonate (CaCO_3_) and dolomite (MgCO_3_)), and from decomposition of soil organic C(SOC) by altering microbial communities and activity [19-21]. To date, few studies have evaluated the change of SIC and its importance to soil C sequestration within N addition experiments, despite the abundance of SIC in semi-arid regions [22,23]. Inner Mongolian grassland, under semi-arid climate, is an important terrestrial ecosystem in northern China. Increasing N deposition may have a large influence on SOC dynamics and SIC storage, thus on the capacity of C sequestration in the Inner Mongolian grasslands.

In this paper, we used a seven-year field experiment, comprising a 6-level N addition gradient and 6-level P addition gradient to investigate the effects of N and P addition on soil C sequestration in Inner Mongolian grasslands. The main objectives were to: (1) explore the effect of N and P on soil C sequestration; and (2) quantify the role of SIC in soil C sequestration for under scenarios of increased N deposition.

## Materials and Methods

### Study site

This study was conducted in a typical steppe ecosystem at the Inner Mongolia Grassland Ecosystem Research Station (IMGERS) of Chinese Academy of Sciences. The region had a semi-arid continental climate with mean annual precipitation of 345mm and mean annual temperature of 1.1°C over the period 1980 to 2010 [15]. The soil was classified as dark chestnut (calcic chernozem according to ISSS Working Group RB, 1998) or loamy sand in terms of texture. The experimental plot was located at 43°33′01″N, 116°40′20″E at an average elevation of 1200 m above sea level. The plot has been fenced off since 1999 to prevent grazing and trampling by large animals (e.g., sheep, cattle, and horses) [16].

### Experimental design

Detailed information of the experimental design used in this research was reported in previous studies [24,25]. In brief, the field experiments for N (urea) and P (potassium phosphate) addition have been conducted in a *Leymus chinensis* grassland since 2006, where the predominant species were *L. chinensis*, *Stipa grandis*, *Cleistogenes squarrosa*, and *Agropyron michnoi* N addition regimes were designated as control (CK), 0 (N1), 5.6 (N2), 11.2 (N3), 22.4 (N4), 39.2 (N5), or 56(N6) g N m^-2^ yr^-1^. Similarly, P addition regimes were designed as control (CK), 0 (P1), 1.55 (P2), 3.1(P3), 6.2 (P4), 9.3 (P5), or 12.4 (P6) g P m^-2^ yr^-1^. Other than in the control, 1.55 g P m^-2^ was also added to each plot in the N addition experiment, and 2.8 g N m^-2^ was added to each plot in the P addition experiment. Thus, there were seven levels for both N and P application with six replicates, giving a total of 84 experimental plots (6 m × 6 m)(Figure S1 in File S1). The fertilizer was thoroughly mixed with sand and then applied in late May, from 2006 to 2012. Our experimental design provided two unique series, one being a N-addition gradient without P limitation, and the other being a P-addition gradient without N limitation.

### Field sampling

At the end of July 2012, we established one sampling quadrat (0.5 m × 1.0 m) in each plot. We first investigated aboveground biomass (AB) with all plant species combined. Litter was subsequently collected. Root biomass was determined using a soil corer (diameter, 7 cm). The samples were collected separately from 5-points within each sampling quadrat, at 0–10 cm, 10–30 cm, 30–50 cm, and 50–100 cm in each plot. Similarly, soil samples were collected using a soil auger (4 cm in diameter) at 0–10 cm, 10–30 cm, 30–50 cm, and 50–100 cm.Bulk density of each soil layer was measured by the IMGERS, using the core method (volume 100 cm^3^) with3 replicates.

### Chemical analysis

Plant, litter, root, and soil samples were ground using a ball mill (M400, Retsch, Germany). The concentration of soil total carbon (STC) was measured by dry combustion using an elemental analyzer (VARIO MAX CN, Elementar, Hanau, Germany). The concentration of SIC was measured by manometric collection of CO_2_ evolved during an HCl treatment process. SOC was calculated as the difference between STC and SIC. C concentrations in plant, root, and litter were measured using the elemental analyzer. Soil pH was determined via a pH meter using soil mixed with distilled water (ratio 1:2.5).

### Calculations and statistical analysis

STC, SIC, and SOC (g C m^-2^) were calculated on an area basis to a soil depth of 100 cm, as described previously [15]:

$$\text{STC}=\sum D_{i}\times S\times B_{i}\times OM_{i}\times 10$$

where D_*i*_, S, B_*i*_, OM_*i*_, and TN_*i*_ represent the thickness of the soil layer (cm), cross-sectional area (m^2^), bulk density (g cm^-3^), and total C concentration (g kg^-1^), respectively; *i* = 1, 2, 3, and 4.

Moreover, the differences in STC and SOC storage between N or P treatments and CK plots was calculated, and was used as the capacity of soil C sequestration.

One-way analysis of variance (ANOVA) was used to determine the effects of N and P addition on the C storage in AB, litter, roots, and soil. Regression analyses were used to test the relationships between the storage of STC, SOC, and SIC and the addition intensities of N or P. Data were represented as mean ±1 standard deviation (n = 6). All analyses were conducted using SPSS statistical software (ver. 13.0, SPSS, Chicago, IL, USA).

## Results

### Changes in plant and soil C storage

**Figure 1:**
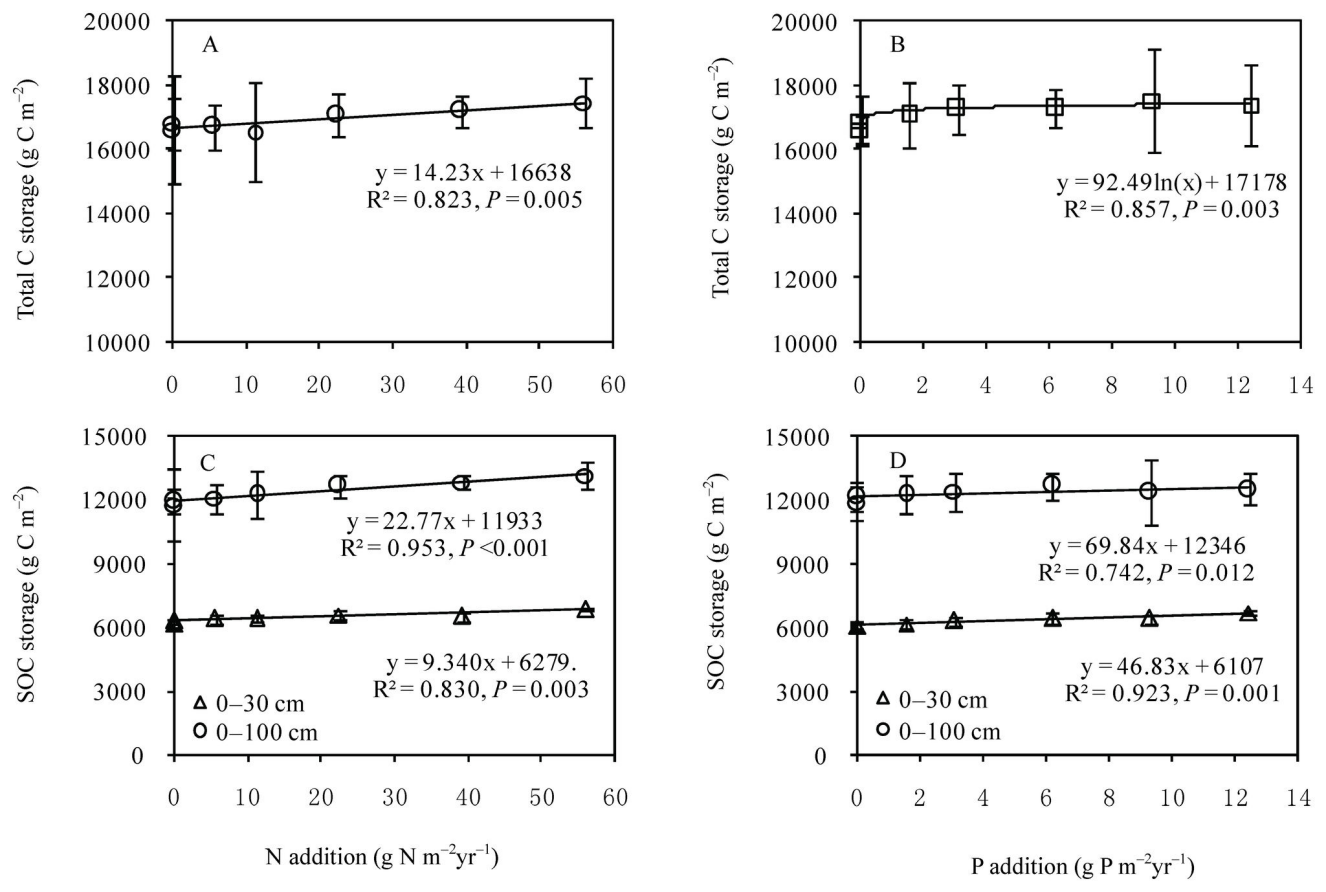
Changes in the carbon storage of Inner Mongolian grasslands with N addition (A and C) and P addition (B and D). Total C storage including C storage of ANPP, litter, roots, SOC, SIC (i.e. top 100-soil layer). Data are presented as mean ± 1 SD (n = 6). See Table 1 for N and P addition and abbreviations.

C stored in AB and litter significantly increased with increasing N addition (F = 3.89, *P* = 0.04 for AB; F = 7.33, *P* < 0.001 for litter) (Table 1), but no apparent effects were observed for P addition. In comparison with soil and roots, C storage in AB and litter was negligible (<1%). C storage in roots varied from 617.4 to 699.7 g C m^-2^ in the 0–100-cm soil layer (Table 2), but did not increase significantly with addition of N and P (Table 1). C storage in grasslands (including AB, litter, root, SOC, and SIC in the 0–100-cm soil layer) varied from 16615 to 17448 g C m^-2^ for the N-addition series, and from 16628 to 17538 g C m^-2^ for the P-addition series, but no significant differences were observed. However, C storage in grassland increased linearly with the intensity of N input (R^2^ = 0.82, *P* = 0.005), and increased quadratically with P addition intensity (R^2^ = 0.86, *P* = 0.003) (Figure 1).

**Table 1:** Changes of C storage in Inner Mongolian grasslands with N and P addition.

	C storage (g C m^-2^)				
	Aboveground biomass	Litter	Roots (0–100 cm)	SOC (0–100 cm)	SIC (0–100 cm)
**N addition**					
CK^†^	40.4±3.6^a^	31.8±7.6^a^	620.1±85.5^a^	11770±1684^a^	4339±111^a^
N1	44.3±8.5^ab^	31.5±7.4^a^	635.7 ±65.0^a^	11926±554^a^	4355±706^a^
N2	46.4±10.2^ab^	33.9±7.2^a^	639.8 ±84.6^a^	12072±674^a^	4108±202^ab^
N3	44.2±7.7^ab^	49.2±10.6^b^	658.5 ±82.6^a^	12254±1093^a^	3725±460^b^
N4	53.0±10.6^bc^	46.2±6.6^bc^	673.8 ±35.3^a^	12620±569^a^	3876±192^ab^
N5	62.6±12.7^c^	39.8±6.2^ac^	698.5 ±96.8^a^	12826±347^a^	3778±404^a^
N6	60.4±16.8^c^	49.4±3.2^b^	699.7 ±70.5^a^	13122±637^a^	3715±524^a^
F-value	3.89 (0.04)	7.33 (<0.001)	0.99 (0.444)	1.96(0.099)	2.67(0.031)
**P addition**					
CK	40.7±10.6^a^	31.7±8.2^a^	617.4 ±59.5^a^	11882±808^a^	4266±591^a^
P1	45.2±5.6^a^	33.7±6.0^a^	647.6 ±46.4^a^	12176±675^a^	4221±675^a^
P2	46.9±7.0 ^a^	32.4±5.0^a^	650.0 ±106.8^a^	12294±926^a^	4363±935^a^
P3	48.4±11.6^a^	33.9±2.2^a^	662.6 ±68.8^a^	12423±909^a^	4305±251^a^
P4	46.0±10.7^a^	33.8±7.9^a^	671.6 ±94.3^a^	12671±634^a^	4089±298^a^
P5	45.8±9.9^a^	33.3±7.2^a^	671.9 ±48.9^a^	12405±1519^a^	4303±424^a^
P6	46.7±6.5^a^	35.2±4.9^a^	679.6 ±102.6^a^	12497±740^a^	4350±550^a^
F-value	0.424 (0.858)	0.207 (0.972)	0.43(0.853)	0.38(0.885)	0.97(0.463)

^†^ CK, control; N1, 0 g N m^-2^ yr^-1^, N2, 5.6 g N m^-2^ yr^-1^; N3, 11.2 g N m^-2^ yr^-1^; N4, 22.4 g N m^-2^ yr^-1^; N5, 39.2 g N m^-2^ yr^-1^; N6, 56 g N m^-2^ yr^-1^; P1, 0 g P m^– 2^ yr^-1^; P2, 1.55 g P m^– 2^ yr^-1^; P3, 3.10 g P m^– 2^ yr^-1^; P4, 6.20 g P m^– 2^ yr^-1^; P5, 9.30 g P m^– 2^ yr^-1^; P6, 12.4 g P N m^-2^ yr^-1^.

^‡^ Data are presented as mean ± 1 SD (n = 6), and those designated with the same letters are not significantly different (*P* <0.05).

**Table 2:** C storage in roots within the different soil layers

	Carbon storage in roots (g C m^-2^)			
	0–10 cm	10–30 cm	30–50 cm	50–100 cm
**N addition**				
CK	232.6±81.4^ab^	202.9±20.6^a^	103.8±12.5^a^	80.8±25.8^a^
N1	234.4±45.3^ab^	228.2±24.7^a^	98.1±18.7^a^	75.1±24.8^a^
N2	202.0±25.5^ab^	231.9±64.5^a^	105.8±20.9^a^	100.1±38.2^a^
N3	244.2±54.5^ab^	217.1±27.4^a^	118.4±44.4^a^	78.8±24.4^a^
N4	272.2±23.6^ab^	214.6±19.3^a^	104.0±12.0^a^	83.0±7.8^a^
N5	269.3±43.5^a^	205.3±31.2^a^	123.7±22.1^a^	100.3±29.6^a^
N6	304.3±47.8^b^	197.5±48.6^a^	104.8±18.8^a^	93.2±15.0
**P addition**				
CK	234.2±17.6^a^	173.9±43.8^a^	113.3±36.0^a^	95.9±12.6^a^
P1	235.4±34.3^a^	198.4±30.8^a^	110.7±13.6^a^	103.1±12.5^a^
P2	217.0±35.4^a^	235.0±44.1^a^	115.4±22.2^a^	82.6±41.8^a^
P3	258.8±26.7^a^	218.4±59.4^a^	103.3±19.5^a^	82.1±17.4^a^
P4	245.2±45.0^a^	220.5±70.0^a^	113.0±21.6^a^	93.0±10.0^a^
P5	246.5±56.4^a^	198.4±28.6^a^	132.6±11.0^a^	94.4±19.9^a^
P6	267.3±46.9^a^	191.6±81.5^a^	136.9±9.1^a^	83.9±14.3^a^

Data are presented as mean ± 1 SD (n = 6), and those designated with the same letters are not significantly different (*P* <0.05).

### Changes in SOC and SIC

SOC in the 0–100-cm soil layer ranged from 11770 to 13122 g C m^-2^ with N addition (Table 1, Table 3), and there was a strong, positive linear relationship with the intensity of N input within the 0–30 cm (R^2^ =0.83, *P* = 0.003) and 0–100 cm soil layers (R^2^ = 0.95, *P* <0.001) (Figure 1). Similarly, SOC increased quadratically with the intensity of P input in the 0-30cm (R^2^ = 0.92, *P* = 0.001) and 0–100 cm (R^2^ =0.74, *P* = 0.012) soil layers (Figure 1).

**Figure 2:**
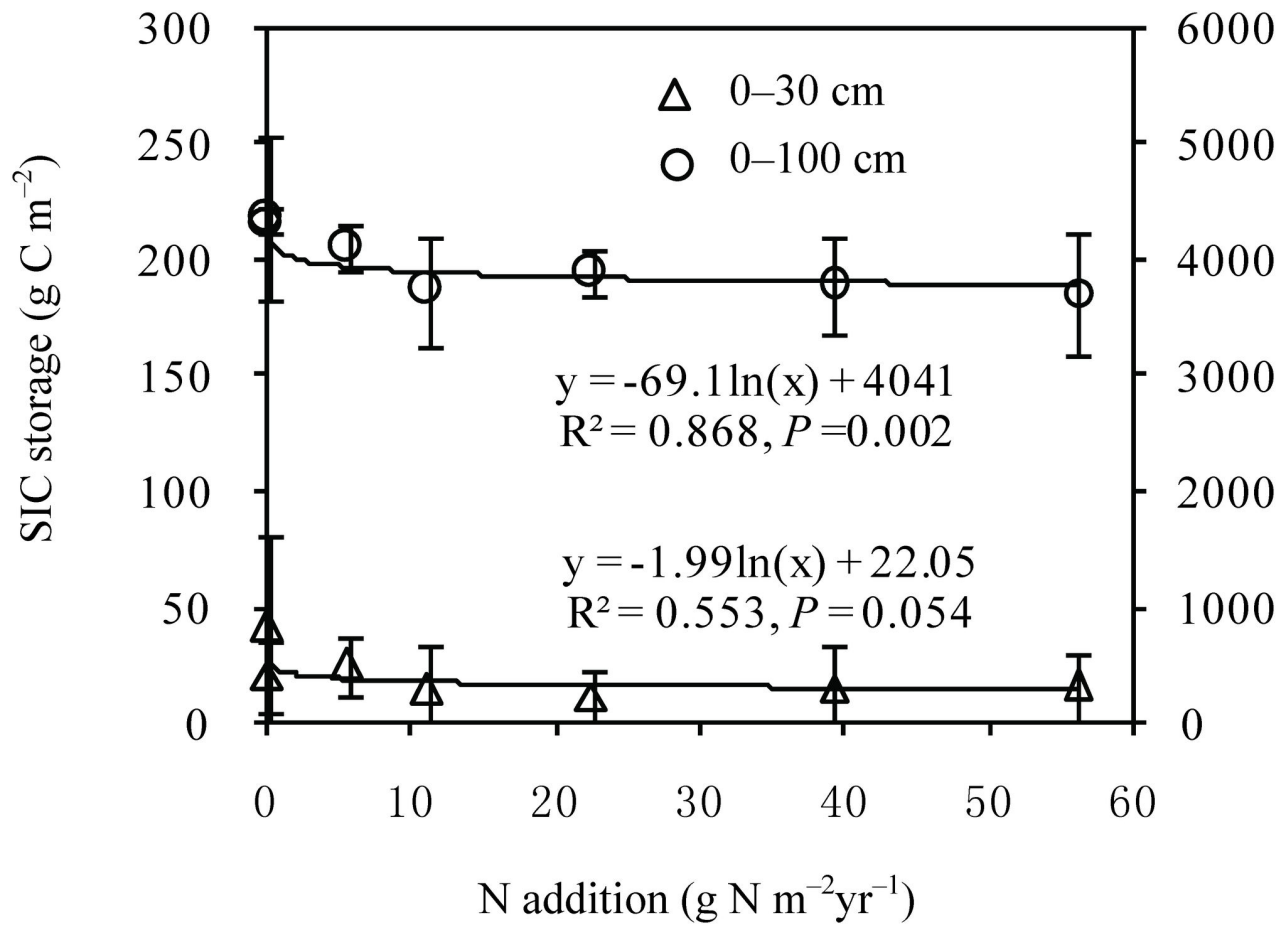
Changes in SIC within 0–30 cm and 0–100 cm soil layers with N addition.

 In response to N addition, soil pH decreased from 7.0 to 6.0 in the 0–10-cm soil layer and from 7.5 to 7.2 in the 10–30-cm soil layer (Table S1 in File S1). SIC in the 0–30-cm soil layer differed significantly between N treatments (F = 3.92, *P* <0.05), but there were no significant differences in the 0–100 cm soil layer (Figure 2). Regression analyses showed that SIC decreased quadratically in the 0–30 cm (R^2^ = 0.55, *P* = 0.05) and 0-100-cm soil layers (R^2^ = 0.87, *P* = 0.002) with increasing N addition (Table S2 in File S1). P addition had no apparent effect on SIC storage.

**Table 3:** Changes in soil organic carbon (SOC) storage within the different soil layers.

	SOC storage (g C m^-2^)			
	0–10 cm	10–30 cm	30–50 cm	50–100 cm
**N addition**				
CK	2611±101 ^a^	3492±125 ^a^	2370±180 ^a^	3256±344 ^a^
N1	2594±119 ^a^	3563±235 ^a^	2499±323 ^a^	3270±564 ^a^
N2	2627±20 ^ab^	3761±212 ^a^	2314±330 ^a^	3370±296 ^a^
N3	2715±128 ^b^	3705±152 ^a^	2337±305 ^a^	3378±321 ^a^
N4	2878±65 ^c^	3709±233 ^a^	2647±310 ^a^	3350±46 ^a^
N5	2852±83 ^c^	3712±233 ^a^	2653±166 ^a^	3347±460 ^a^
N6	2946±108 ^c^	3997±238 ^b^	2719±278 ^a^	3397±904 ^a^
**P addition**				
CK	2579±148 ^a^	3486±463 ^a^	2518±111 ^a^	3300±327 ^a^
P1	2596±61 ^a^	3487±276 ^a^	2568±343 ^a^	3690±449 ^a^
P2	2678±220 ^b^	3518±313 ^a^	2524±327 ^a^	3692±334 ^a^
P3	2833±111 ^b^	3497±387 ^a^	2620±464 ^a^	3473±301 ^a^
P4	2816±149 ^b^	3631±282 ^a^	2561±277 ^a^	3663±352 ^a^
P5	2788±80 ^b^	3642±446 ^a^	2452±284 ^a^	3522±519 ^a^
P6	2833±133 ^b^	3695±528 ^a^	2495±439 ^a^	3475±290 ^a^

Data are presented as mean ± 1 SD (n = 6), and those designated with the same letters are not significantly different (*P* <0.05).

### Effects of N and P addition on soil C sequestration

**Figure 3:**
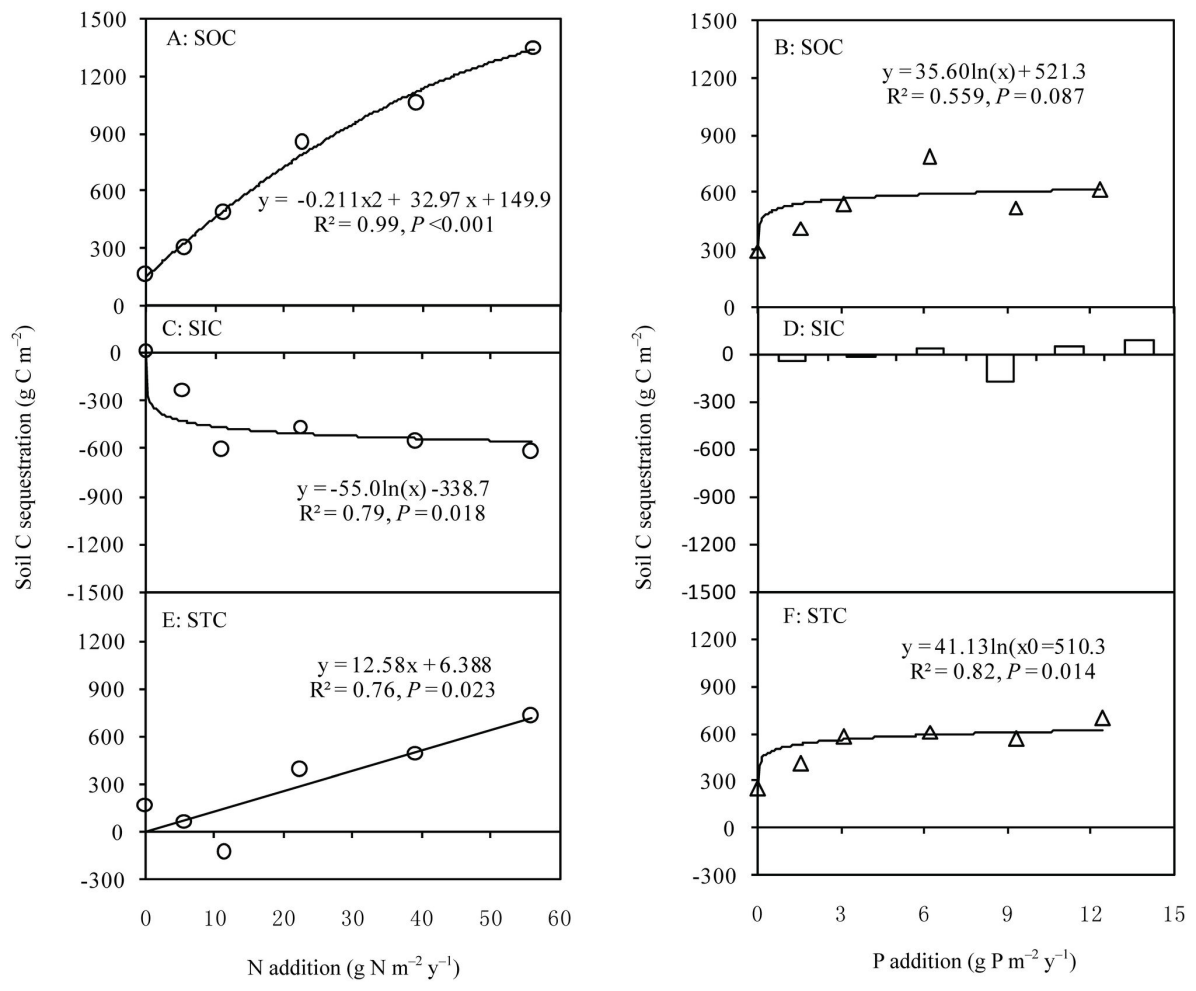
Soil C sequestration of grasslands in the 0–100 cm soil layer with N addition and P addition. STC, soil total carbon; SOC, soil organic carbon; SIC, soil inorganic carbon.

**Figure 4:**
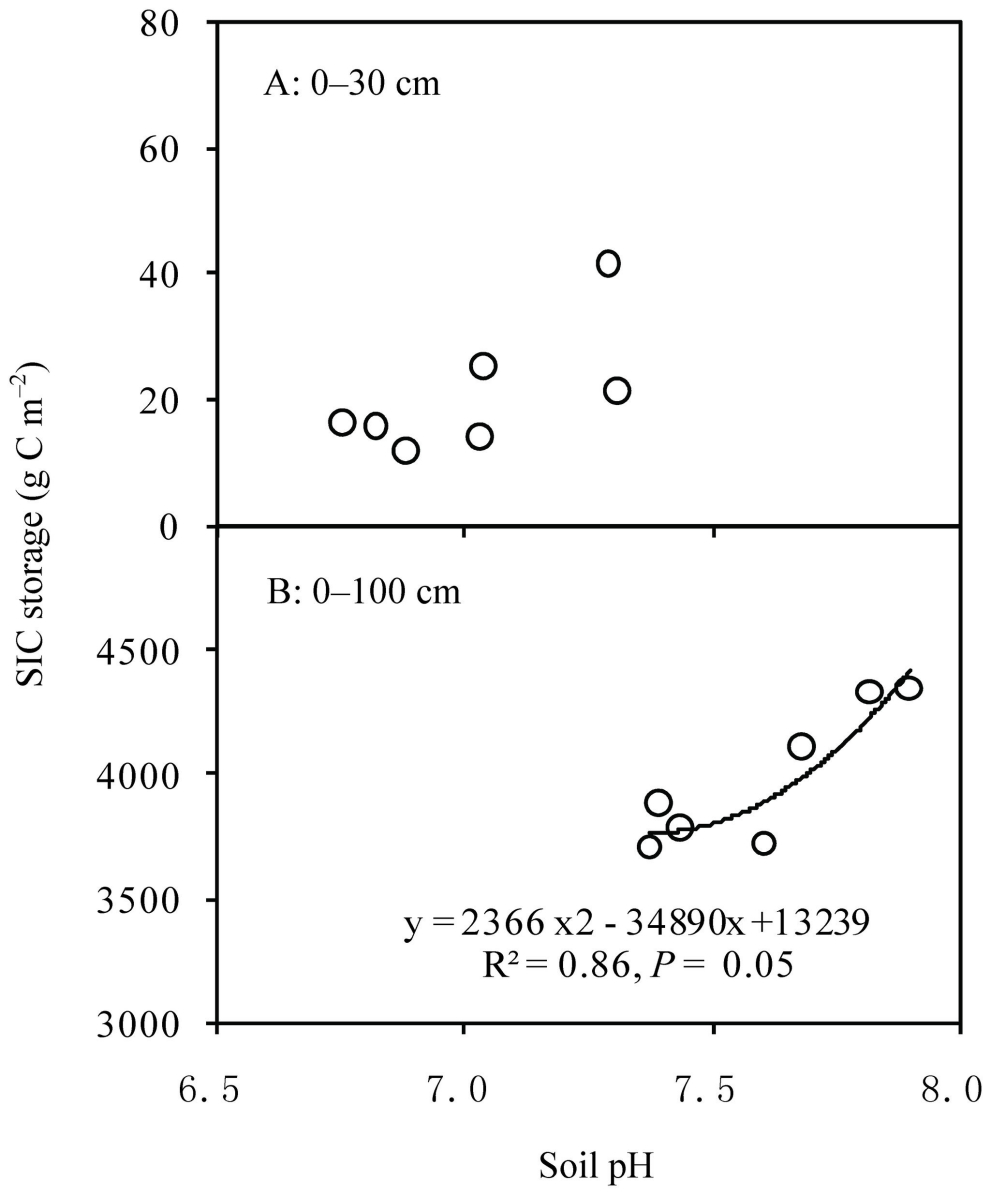
Relationship between SIC and soil pH in response to N addition.

**Figure 5:**
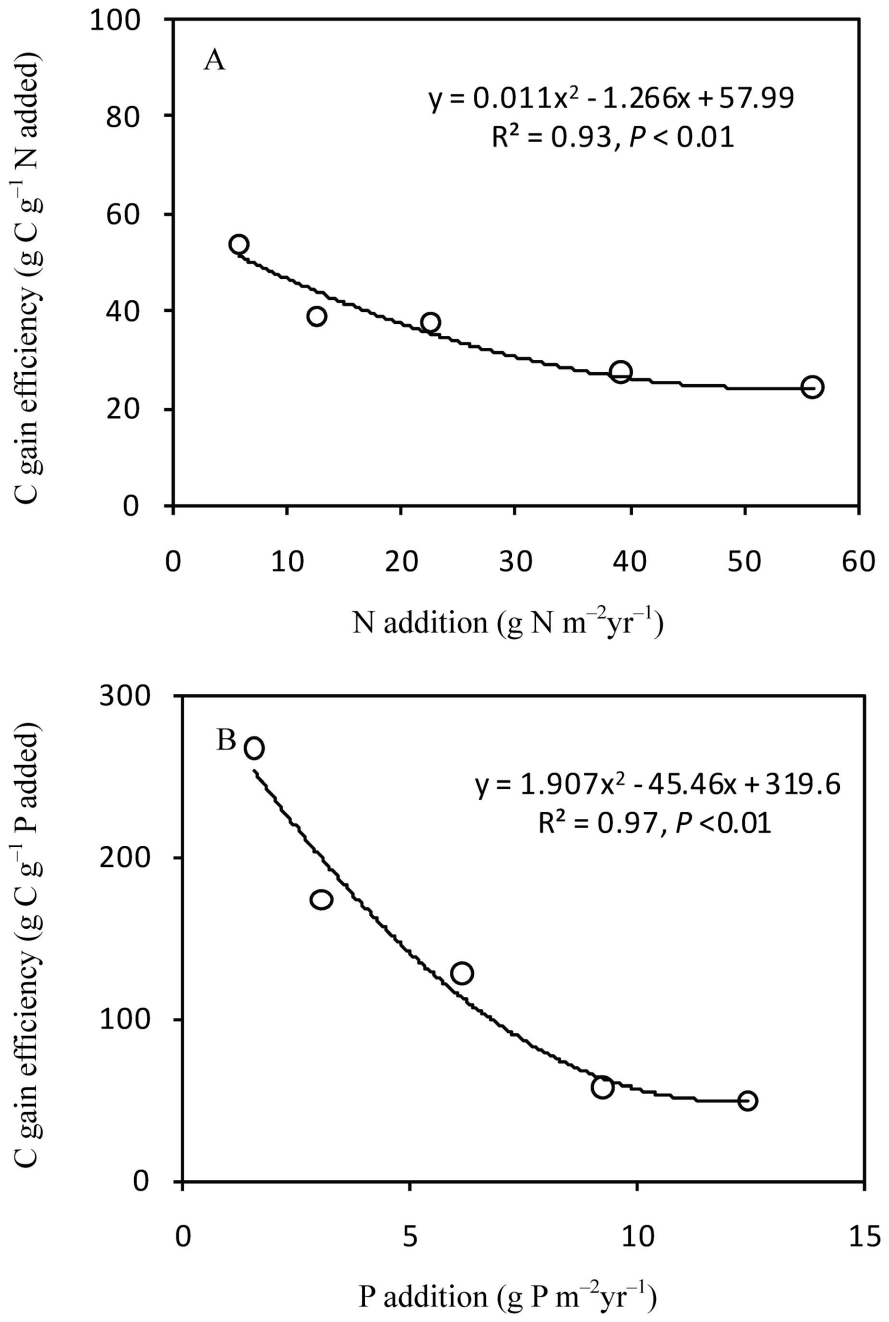
C gain efficiency of SOC under N addition (A) and P addition (B)

C sequestration by SOC content in the 0–100-cm soil layer ranged from 156 g C m^-2^ in N1 to 1352 g C m^-2^ in N6, and increased quadratically with the intensity of N input (R^2^ = 0.99, *P* < 0.001; Figure 3). However, SIC storage decreased logarithmically with increasing N addition (R^2^ = 0.79, *P* = 0.018), which was negatively correlated with soil pH (Figure 4). C sequestration by STC showed significant linear correlation with increasing N addition (R^2^ = 0.76, *P* =0.023). C sequestration by SOC and STC showed logarithmic response to increased P input (R^2^ = 0.56, *P* = 0.087 for STC; R^2^ = 0.82, *P* = 0.014 for STC) (Figure 3). Moreover, the efficiency of C gain decreased with increasing N and P input (Figure 5).

## Discussion

The addition of N enhances C storage in Inner Mongolian grasslands within aboveground biomass, litter, roots and soils. SOC storage in the 0–100 cm soil profile increased quadratically (from 156 to 1352 g C m^-2^) with the intensity of N input. The increase in C input by roots and aboveground biomass was the main reason for the increase in soil C storage (Table 1 and Table 2). Fornara and Tilman [26] found that the total ecosystem C storage increased significantly after 27 years of increasing N input, and suggesting N-induced increase in root mass and community transformation as the potential mechanisms. Roots make substantial C contributions to mineral soils [27], mainly through root turnover and decomposition but also through root exudation [14]. Compared with litter, roots are more important sources of new organic matter in Inner Mongolian grasslands [15]. Moreover, C gain efficiency decreased quadratically with increasing N and P addition in Inner Mongolian grasslands (Figure 5), similar to the findings in the prairie grasslands in the USA [26] and forests in Sweden and Finland [28]. Therefore, it is important to further investigate the underlying mechanisms of decreasing C gain efficiency in the future.

The questions of where, how, and if N addition enhances soil C sequestration in terrestrial ecosystems remain controversial [14,29]. Through a 5-year N-addition experiment (20 g N m^-2^), Zeng et al. [13] found that the C storage of roots and SOC in Keerqin sandy grasslands decreased by 84.8 and 128.5 g C m^-2^, respectively, although aboveground biomass and litter increased. In a 2-year N-addition experiment (17.5 g N m^-2^), Lu et al. [30] reported that the increase in C storage was 18.7, 8.7, and 377.9 g C m^-2^ in aboveground biomass, litter, and the 0–40 cm soil layer, respectively, but decreased by 90 g C m^-2^ in roots. Lu et al. [14] conducted a meta-analysis of 257 published studies and found that N addition had no significant effect on soil C storage in forests and grasslands, although N addition enhanced C inputs from vegetation to soil. Waldrop [31] found that soil C storage decreased significantly (20%) in a sugar maple-dominated ecosystem but increased significantly (10%) in an oak-dominated ecosystem under 3-year N addition. Based on our findings, we conclude that Inner Mongolian grasslands have an apparent potential to sequester atmospheric CO_2_ in soil for scenarios of increasing N deposition. 

P addition also enhanced soil C sequestration in semi-arid grasslands, but the increase showed a logarithmic relationship to the intensity of P input. This founding implied a lower saturation level for P in terms of C sequestration. C gain efficiency decreased quadratically with increasing P input, which also supported this assumption. The possible explanation for the observed logarithmic relationship is that P absorption proficiency of plant species decreased in response to P addition intensities[32]. Niu et al. [33] reported that P addition has no apparent effect on net ecosystem exchange in temperate steppe. Based on the increase and trend observed for SOC, we suggest that N addition will have a greater effect than P addition in Inner Mongolian grasslands. Moreover, stoichiometry can provide a new approach for discussing the constraining effect of N and P on C sequestration [34], because the N:P ratio of soil organic matter (SOM) in Inner Mongolian grassland is constrained within a rather narrow range, with the C:N:P ratio being 98:6:1 for the experimental plot [24,35]. 

A decrease in soil pH is commonly observed with increasing reactive N input (N deposition or N addition experiments)[19-21]. Most studies have emphasized the regulatory effect of soil pH on SOM turnover due to the alternation of microbial communities and activity [9,36], but have ignored the effect of soil acidification on SIC. In this study, the soil pH decreased by 0.4–1.0 units in the surface soil layer and was significantly correlated with the intensity of N input (*P* <0.001). However, there were no apparent changes in pH within deeper soil profile during this 7-year N-addition experiment. However, a longer field experiment may be required to observe effects of N addition on pH in deeper soil layers. Soil pH after N addition depends on the balance between acid and non-acid cations on colloid surfaces and the balance between hydrogen (H^+^) and hydroxide (OH^-^) ions in soil solution. Theoretically, if all of the ammonium ions from ammonium sulfate are nitrified then 1 mol ammonium sulfate produces 4 mol acid (+). Thus, the application of 24 kg N ha^-1^ as ammonium sulfate requires nearly 165 kg CaCO_3_ ha^-1^ to neutralize it [37]. Along with increasing reactive N input (N fertilizer or increasing atmospheric N deposition), croplands and grasslands in China showed significant acidification from the 1980s to the 2000s [21,38]. 

Soil acidification could induce substantial CO_2_ emission from dissolution of carbonates (such as calcium carbonate (CaCO_3_) and dolomite (MgCO_3_)) in semi-arid Inner Mongolian grasslands because of the high SIC content [22,23]. Our results showed that SIC decreased from 230.8 to 623.9 g C m^-2^ in the 0–100-cm soil layer in Inner Mongolian grasslands, and that the decreases in SIC could offset 46–76% of the increases in SOC associated with N addition. Yang et al. [23] estimated that, with a decrease 0.63 in the soil pH, SIC storage in the top 10 cm decreased by an average of 26.8 g C m^−2^ yr^−1^ in northern China. However, a few studies suggest that carbonates may be dissolved in surface soil (through the release of CO_2_fromdecomposition of soil organic matter or by the input of hydrogen ions from acid deposition) but re-precipitated in deep soil layers [23,38,39]. The relationship between soil acidification and SIC storage could be very important for the carbon cycle in semi-arid grasslands in northern China[22]. Therefore, future studies should also include assessment of SIC dynamics in order to accurately evaluate soil C sequestration under N deposition scenarios in semi-arid regions.

## Conclusion

In the semi-arid Inner Mongolian grasslands, soil C sequestration increased quadratically with increasing N input due to the significant increase in litter and root C input; but C gain efficiency decreased with increasing N input. Soil C sequestration increased logarithmically with the intensity of P input and showed a lower saturation level for P than for N. Soil pH apparently decreased with N addition, which resulted in a loss of SIC of 230.8 to 623.9 g C m^-2^ in the 0–100-cm soil layer. Our findings demonstrated that loss of SIC could partially offset the increase in SOC associated with N addition. Therefore, future evaluation of soil C sequestration for scenarios of increasing N deposition should include changes in SIC storage, especially in semi-arid regions.

## Supporting Information

File S1 **Figure S1, Table S1 and Table S2.**
(DOCX)
